# Methylation of a newly identified region of the INS-IGF2 gene determines IGF2 expression in breast cancer tumors and in breast cancer cells

**DOI:** 10.18632/oncotarget.27655

**Published:** 2020-11-03

**Authors:** Vinodh Kumar Radhakrishnan, Kameswaran Ravichandran, Chibuzo Eke, Amanda Ortiz-Vicil, Qianwei Tan, Marino De León, Daisy D. De León

**Affiliations:** ^1^ Center for Health Disparities and Molecular Medicine, Loma Linda University School of Medicine, Loma Linda, CA 92350, USA; ^2^ Division of Renal Diseases and Hypertension, University of Colorado at Denver, Aurora, CO 80045, USA

**Keywords:** IGF2, INS-IGF2, hypermethylation, DMR, epigenetics

## Abstract

IGF2 is essential in breast differentiation, lactation, tumor growth, and in breast cancer (BC) development and progression. This growth factor also inhibits apoptosis and promotes metastasis and chemoresistance, contributing to more aggressive tumors. We previously demonstrated that IGF2 protein levels are higher in BC tissues from African American women than in Caucasian women. We also showed that high IGF2 protein levels are expressed in normal breast tissues of African American women while little or no IGF2 was detected in tissues from Caucasian women. Others showed that decreased DNA methylation of the IGF2 gene leads to different BC clinical features. Thus, we designed this study to determine if differentially methylated regions of the IGF2 gene correspond to IGF2 protein expression in paired (Normal/Tumor) breast tissues and in BC cell lines. Methylation analysis was performed using Sodium Bisulphite Analysis and Methylation Sensitive Restriction Enzyme digestion methods. Our results show that a unique site in the INS-IGF2 region is hypermethylated in normal breast and hypomethylated in breast cancer. We designated this region the DVDMR. Furthermore, the methylation levels in the DVDMR significantly correlated with IGF2 protein levels. This novel DMR consists of 257bp localized in the INS-IGF2 gene. We propose that methylation of DVDMR represents a novel epigenetic biomarker that determines the levels of IGF2 protein expression in breast cancer. Since IGF2 promotes metastasis and chemoresistance, we propose that IGF2 levels contribute to BC aggressiveness. Validation of IGF2 as a biomarker will improve diagnosis and treatment of BC patients.

## INTRODUCTION

Insulin-Like Growth factor 2 (IGF2) is a fetal growth factor that plays a critical function in fetal differentiation and metabolism by signaling through the IGF-I receptor and the insulin receptor [1]. Posttranscriptional processing of the IGF2 protein produces several isoforms including proIGF2 (10-21kD; predominant form in fetal development) and the mature, processed IGF2 (7.5kD; mIGF2) detected in circulation. When breast cancer (BC) develops, proIGF2 form is re-expressed and it promotes tumor growth, chemoresistance and metastasis. IGF2 is inhibited by tumor suppressor genes such as p53 and Pten, both important in normal and in BC development [2, 3]. Lack of expression or mutations in tumor suppressors contribute to higher IGF2 levels, not only for lack of suppression but also for gain of function such as in the case of mutated p53 that stimulates IGF2 instead of repressing it [4].

IGF2 expression is increased by Growth Hormone, estrogen, progesterone and prolactin, all important hormones in normal breast development and in BC progression [5–7]. Furthermore, “free” circulating IGF2 levels in humans are significantly correlated to breast tumor size and malignancy [8]. Thus, IGF2 expression is important in normal breast development and increased IGF2 expression in the mammary gland contributes to BC malignancy.

IGF2 is one of the best characterized epigenetically modified loci in the human genome. This gene is located on the short (p) arm of chromosome 11 at position 15.5. Methylation of the IGF2 gene regulatory regions occurs during the formation of an egg or sperm cell, and it is distinct and differentially modified depending on the parental origin of the allele [9, 10]. The IGF2 gene has five promoters (P0, P1-P4) that generate distinct transcripts which vary by tissue type and developmental stages [11, 12]. In normal human tissues, the IGF2 gene is controlled by at least two differentially methylated regions (DMRs). One DMR is located upstream of the IGF2 promoters (IGF2 DMR) and the second is located upstream of the neighboring non-coding H19 gene (H19 DMR) [13]. Shifts in methylation established at these DMRs can lead to loss of imprinting, causing increased transcription and translation of IGF2.

When normal physiological mechanisms or homeostasis in the body are affected during embryonic development chronic diseases may develop later in life [13, 14]. Indeed, several epidemiological studies have suggested that risks for adult diseases are associated with adverse environmental conditions experienced in early embryonic development [15, 16]. Thus, chronic diseases affecting adults may be caused by epigenetic changes that modify the fetal DNA, and this increased risk is heritable by the altered DNA methylation that controls specific genes.

The epigenetic information of the IGF2 gene is stored via heritable DNA methylation [14, 17]. In addition, the organization of the IGF2 gene chromatin structure and its regulatory elements involves various long linear non-coding RNA and microRNAs at the early embryonic developmental stages [18–22]. Thus, the IGF2 DMR and the CpG island regions play a vital function in early embryonic development, and its methylation status is subject to modification by fetal environmental exposures such as famine, nutrition and stress [15, 23]. Abnormal regulation of IGF2 leads to various types of cancers and metabolic disorders like BC, pancreatic cancer, diabetes and endocrine related disorders [24–26]. In particular, dysregulation in the methylation of the IGF2 gene promoters occurs in several cancers including BC [27] and this altered methylation leads to different clinical features in BC disease [28].

In spite of these advances, there is currently no consensus regarding the methylation status of the IGF2 gene, and its relationship to the levels of IGF2 protein expressed in normal breast or in breast cancer tissues. To address this need, the present study focused on the DNA methylation patterns of the IGF2 gene in paired (normal/tumor) tissues obtained from African American (AA), Caucasian (CA), South Korean (SK) and Vietnamese (VIET) BC patients. DNA methylation patterns of the IGF2 gene were also analyzed in several BC cell lines to determine if there was a correlation between methylation of the IGF2 gene regulatory regions and the cellular expression levels of IGF2 protein.

## RESULTS

### Identification of a novel differentially methylated region that controls the IGF2 gene

To assess if DNA methylation in the IGF2 gene correlated with protein expression, we used specifically designed primers to analyze methylation levels in well-known regulatory regions of the IGF2 gene as detailed in Table 1. We analyzed methylation levels in four CpG islands, two differentially methylated regions (DMRs), and four IGF2 gene promoters. In addition, we also designed dozens of primers to test for potentially new DMRs within the IGF2 gene but upstream and downstream of the regions described above. Only one new DMR was uncovered and the primer used to identify it is shown on Table 1.

**Table 1:** IGF2 primers for CPG islands, DMROs and IGF2 P1-4 promoters

IGF2 Target region	Forward and reverse primers sequence (BPs)
CpG1 (250bp)	(F) 5’- GTTTGGAGTTGGGGTTTGG-3’
	(R) 5’- CTACCTCAATCCCTAAAATC-3’
CpG3 (250bp)	(F) 5’- GATTTTTGGGGGATTTTAATTTAT-3’
	(R) 5’- CCACATCCTAAAAAACCAAAC-3’
CpG4 (250bp)	(F) 5’- GTAGGGGTTTGTTTGTTTTTTTG -3’
	(R) 5’- CTACTATACTTCCTCAACCC -3’
CpG5 (250bp)	(F) 5’- GAAGATGCTGCTGTGCTTCC -3’
	(R) 5’- AGTGAGCAAAACTGCCGC -3’
DMRO1 (250bp)	(F) 5’- TTGGTGTTGGAAAGTGTTTG-3’
	(R) 5’- CTATAACRTCCAAACCCTCTA-3’
DMRO2 (250bp)	(F) 5’- GTTAAGGTAGTTTTTTTGGG-3’
	(R) 5’- AATAACCCRCCTTAAAAAATC-3’
DVDMR (200bp)	(F) 5’- AGGATGGGTTTTTGTTTGGTATT-3’
	(R) 5’- AAAAAAATTCATTTCCCCAAAAA-3’
IGF2 - P1 (172bp)	(F) 5’- ATTACACGCTTTCTGTTTCTCTCC-3’
	(R) 5’- AAATGAGGTCAGCTGTTGTATCAAG-3’
IGF2 - P2 (59bp)	(F) 5’- TGCTTTGGTGGTGACTGCTAA-3’
	(R) 5’- GAAACTGCCTGGACGATGATC-3’
IGF2 -P3 (bp)	(F) 5’- GGTTCCCACATTAACGGAGTC-3’
	(R) 5’- ACGCAAGGCAGAGTTCTTTC-3’
IGF2 -P4 (bp)	(F) 5’- TCTCCTGTGAAAGAGACTTCCAG-3’
	(R) 5’- CTGGTGCTTCTCACCTTCTTG-3’
IGF2 Total (68bp)	(F) 5’- CCGTGCTTCCGGACAACTT -3’
	(R) 5’- CTGCTTCCAGGTGTCATATTGG -3’
Universal Methylated	(F) 5’- GGAGTGAAGGAGGTTACGGGTAAGT-3’
Primer STD (128bp)	(R) 5’- AAAAACGATAAAACCCTATACCTAATCTATC-3’

**Figure 1:**
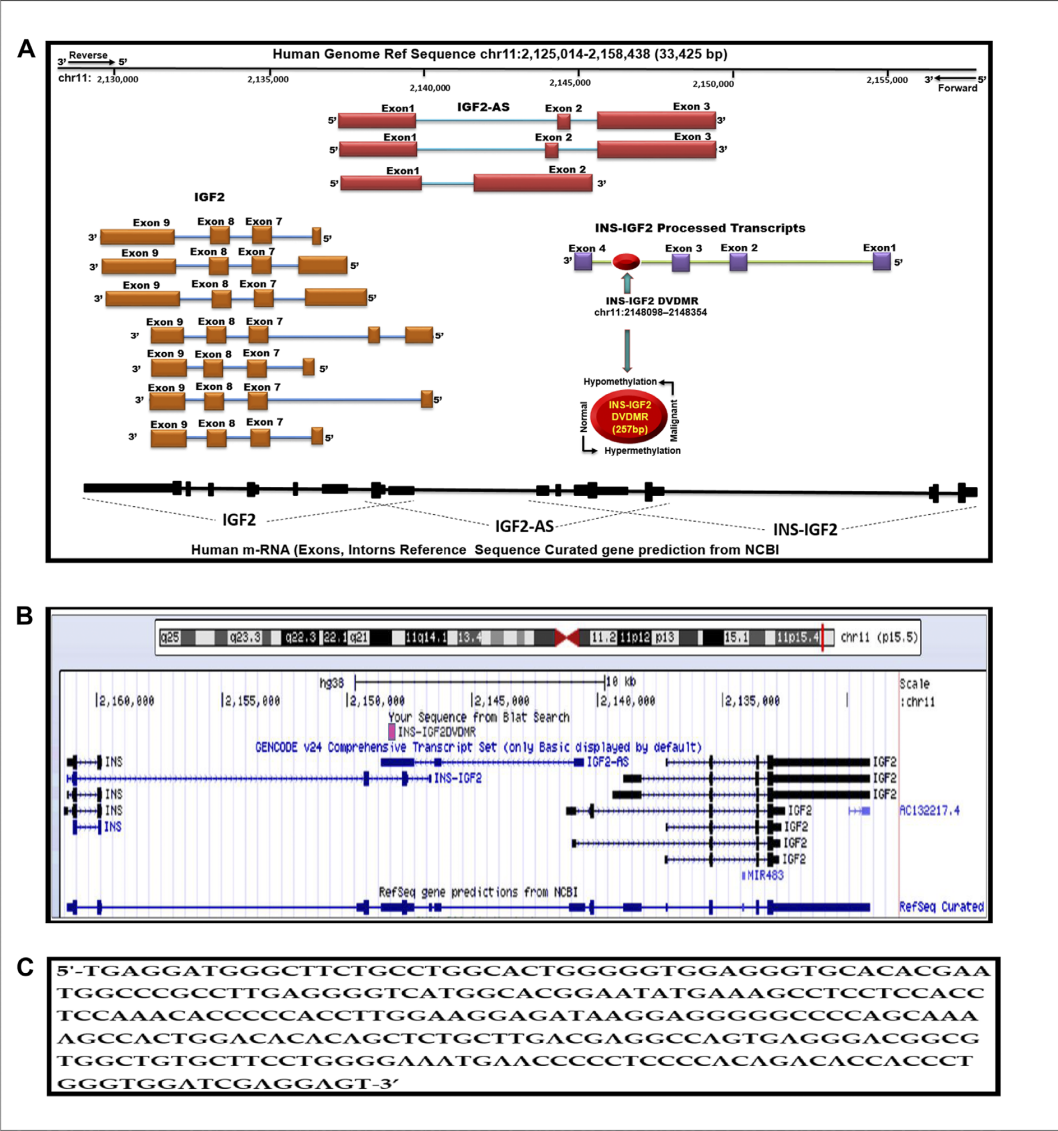
Schematic diagram of the human IGF2 gene structure. (**A**) The IGF2 gene diagram showing the relative positions of the IGF2 introns and coding exons. Also present in this region is the INS-IGF2 and the IGF2AS genes which code for non-translatable mRNAS. The newly identified differentially methylated region (DVDMR) is represented in red (chr11:2148098-2148354). This region is hypermethylated in normal breast tissues and hypomethylated in breast cancer. (**B**) Figure obtained from the UCSC Genome Browser, Dec. 2013 (GRCh38/hg38) showing in pink the DVDMR located between exon 1 and exon 2 of the IGF2 gene showing chr11:2148098 2148354 5′-3′ pad strand = + sequence from Blat search in reference to human genome sequence; (**C**) INS-IGF2 DVDMR BLAT search sequence (chr11:2148098-2148354).

A modified diagram of the IGF2 gene and its transcript variant structure as described in the Human UCSC Genome Bioinformatics browser Dec. 2013 (GRCh38/hg38) Assembly is shown in Figure 1A. Note the curated gene prediction from NCBI for the IGF2 gene including processed exons and intron structure. The IGF2 gene structure is shown to frame the newly identified DMR denoted in the red circle. We named this region the INS-IGF2 DVDMR. The DVDMR (DaisyVin Differentially Methylated Region) is hypermethylated in normal breast tissues and hypomethylated in BC. To the best of our knowledge, this DMR has not been characterized or previously identified. It is located between exons 3 and 4 of the INS-IGF2 (Insulin-IGF2) gene which is part of the IGF2 intergenic region of Chromosome 11.

We lined up all mRNAs known to be transcribed from the IGF2 intergenic region (Figure 1B). Note that the DVDMR is located between exons 3 and 4 of the INS-IGF2 gene (Figure 1A). Furthermore, the DVDMR is also within exon 3 of the IGF2 anti sense (AS). The significance of this observation is not clear at present but it is important to know that both mRNAs (INS-IGF2 and IGF2 AS) are non-coding RNAs that do not encode proteins but are important in the translation of other mRNAs or epigenetic DNA modifications. To determine the sequence of the newly identified INS-IGF2 DVDMR, we used the BLAST tool to search the UCSC Human genome browser. Figure 1C shows the 5′-3′ strand sequence of the 257 base pairs DVDMR, localized in chr11:2148098–2148354.

### Expression of IGF2 mRNA’s from P1-P4 promoters and protein by RPPA in TNBC cells

**Figure 2:**
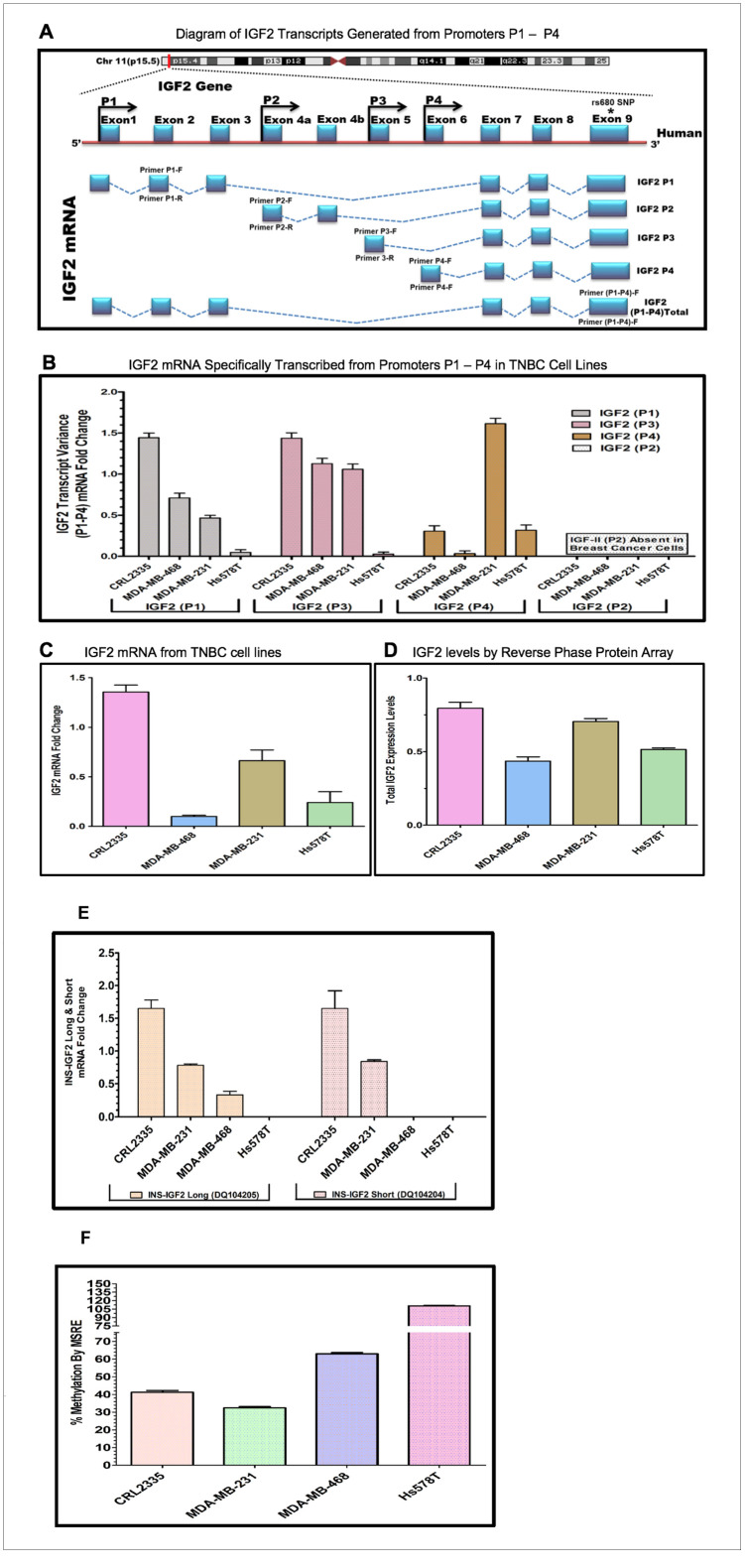
(**A**–**D**) Diagram and bar graphs of the IGF2 gene structure and transcript variance in TNBC cell lines. (A) Diagram showing specific transcripts generated from P1-P4 promoters (modified from references 30, 33). Arrows in P1-P4 promoters indicate the position of the forward and reverse primers designed using intron spanning assay. Capturing of Exon 9 measures the total IGF2 mRNA while the specific designed primers (P1-P4 forward and reverse) accurately measures IGF2 transcripts generated from P1-P4. (B) Shows the specific P1-P4 promoter transcript variance differentially expressed in breast cancer (BC) cell lines; (C) Represents total endogenous IGF2 mRNA fold change expressed in breast cancer cell lines, using QPCR. (D) Bar graph represents the levels of IGF2 protein being expressed in breast cancer cells assessed by Reverse Phase Protein Array (RPPA). (**E**, **F**) Diagram and bar graphs of the INS-IGF2 Transcript Variants (Long and Short). Fig 2E Bar graphs represents the INS-IGF2 Long (DQ104205) and Short (DQ104204) mRNA fold change expressed in African American (AA) and Caucasian (CA) TNBC Cells. Fig 2F. Bar Graph represents the % methylation by Methyl sensitive restriction enzyme assay (MSRE) of newly identified DVDMR region in TNBC cell lines measured using qPCR.

A diagram depicting the IGF2 mRNA splice variants that can be processed from each of the four P1-P4 promoters is shown in Figure 2A. The diagram also shows the specific exons used to develop the primers to identify the IGF2 mRNA generated from each specific promoter (IGF2P1-IGF2P4). In addition, it also shows the primer region developed from Exon 9 which recognizes all combined IGF2 mRNAs.

Initially, we identified the DVDMR utilizing DNA obtained from four different BC cell lines. First, we prepared mRNAs from four TNBC cell lines, two from African American (AA) BC patients (CRL 2335 and MDA 468) and two from (CA) Caucasian patients (Hs578t and MDA-231) (Table 2). Figure 2B shows a representative bar graph of the IGF2 P1-P4 transcript variant fold change analyzed by the intron spanning qPCR method. The predominant IGF2 transcripts were expressed from P1 and P3 except in the Hs578T cells, which produced mRNA predominantly from P4 transcripts while transcripts from P1 and P3 where barely detectable. The TNBC cell line CRL2335 (AA) expressed the highest IGF2 mRNA variant levels from P1 and P3 while the MDA-231 cells expressed high levels of transcripts from P4. No transcripts from P2 were detected in any of the four BC cell lines analyzed. Similar results have been reported in other types of cancers, where IGF2 transcripts were predominantly expressed from P3 while transcripts from P2 were specifically detected in liver [29].

**Table 2:** Paired tissues from AA, CA, VIET breast cancer patients

Sample	ID	Receptor status	Age	Ethnic group
Tumor	1M	ER-/PR-/Her2-	61	AA
Normal	2N			
Tumor	3M	ER-/PR-/Her2-	43	AA
Normal	4N			
Tumor	5M	ER+/PR+/Her2+	58	AA
Normal	6N			
Tumor	7M	ER-/PR-/Her2-	42	AA
Normal	8N			
Tumor	1M	ER-/PR-/Her2+	71	CA
Normal	2N			
Tumor	3M	ER+/PR-/Her2-	62	CA
Normal	4N			
Tumor	5M	ER+/PR+/Her2-	67	CA
Normal	6N			
Tumor	7M	ER-/PR-/ Her2-	80	CA
Normal	8N			
Tumor	1M	ER-/PR-/Her2-	45	VIET
Normal	2N			
Tumor	3M	ER-/PR-/Her2-	47	VIET
Normal	4N			
Tumor	5M	ER-/PR-/Her2-	46	VIET
Normal	6N			
Tumor	7M	ER-/PR-/Her2-	35	VIET
Normal	8N			
Cell Line	CRL2335	ER-/PR-/Her2-	60	AA
Cell Line	MDA-MB-468	ER-/PR-/Her2-	51	AA
Cell Line	Hs578T	ER-/PR-/Her2-	74	AA
Cell Line	MDA-MB-231	ER-/PR-/Her2-	51	CA

We then decided to compare the total IGF2 mRNA levels using a primer that measures all transcripts combined from P1 to P4 (Figure 2C). The levels of total IGF2 mRNA from all P1-P4 promoters demonstrated that the TNBC cell line CRL 2335 (AA) expressed the highest levels of total IGF2 mRNA while the MDA-468 cell line (AA) expressed the lowest total IGF2 mRNA levels. When we compared the levels of total IGF2 mRNA with protein levels, we observed that IGF2 protein levels were comparable in the CRL2335 (AA) and MDA-231cell lines (Figure 2D). Interestingly, the MDA-MB-231 breast cancer cells (CA) expressed the second highest levels of total IGF2 protein even though it represented only 50% of the IGF2 mRNA when compared to the CRL2335 (AA) cell line (Figure 2C). Similarly, cell lines MDA-MB-231 and Hs578t expressed similar amounts of protein but significantly lower levels of IGF2 mRNA. Thus, these results suggest that even when accounting for specific IGF2 promoter activation or measuring total IGF2 mRNA, there is no complete correlation between IGF2 mRNA and IGF2 protein levels. This is due in part to posttranscriptional and posttranslational regulation of both the IGF2 mRNAs and IGF2 protein.

### Relationship of the expression of INS-IGF2 Long and short variant with the INS-IGF2 DVDMR methylation levels by MSRE in breast cancer cells

Since the DVDMR region that controls the IGF2 mRNA is in the INS-IGF2 DNA region, we decided to assess whether methylation of the DVDMR also controls the expression levels of the INS-IGF2 mRNAs. There are two INS-IGF2 mRNAs transcribed from this region which do not code for any protein. We thus, proceeded to compare the methylation levels of the DVDMR region to the expression of the INS-IGF2 mRNA short (DQ104204) and long (DQ104205) transcript variants. As shown in Figure 2E, high levels of the INS-IGF2 long and short transcript variants were detected in the CRL2335 and MDA-231 cells. Of note, both cell lines expressed the highest levels of IGF2 (Figure 2D). Interestingly, the MDA-468 cells only expressed low levels of the long INS-IGF2 transcript variant. In contrast, Hs578T cell line did not express either variant of the INS-IGF2 (Figure 2E). High methylation percentage of the DVDMR region correlated with lower or no expression of the INS-IGF2 transcripts and lower IGF2 protein levels (Figure 2F). By contrast, lower DVDMR methylation levels correlated with higher expression of both INS-IGF2 transcripts and higher IGF2 protein levels.

### Methylation of the IGF2 gene: CpG islands, DMR’s and DVDMR in paired normal/malignant breast tissues

**Figure 3:**
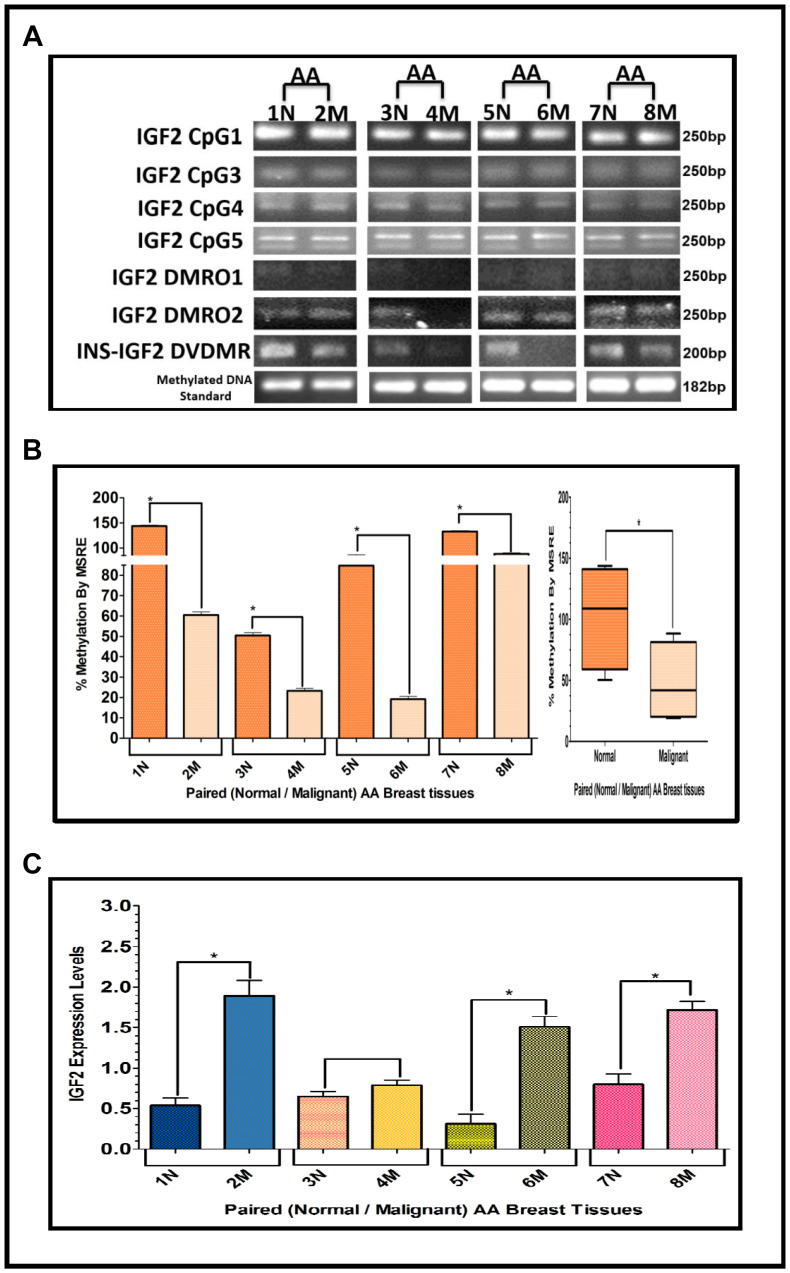
(**A**–**C**) EtBr Agarose gel of IGF2 CpG islands and DMRO’s and Bar graphs of % methylation and IGF2 protein levels in paired normal/malignant breast tissues from AA women. (A) shows one of two ethidium bromide stained agarose gels of Bisulphite g-DNA fragments produced by conventional PCR amplification of the CpG and DMRO regions of the IGF2 gene in paired breast tissues of AA women. (B) Shows a comparative bar graph of the methylation of the INS-IGF2 DVDMR in paired tissues from AA women. Average % MSRE Methylation shown by Box and Whisker Plots. (C) Bar graph shows the IGF2 levels from paired breast tissues from AA women assessed by Reverse Phase Protein Array (RPPA) assay. The details of the paired breast tissue samples shown in Table 2. ^*^symbol denotes paired samples (Normal/Malignant) from same patient.

**Figure 4:**
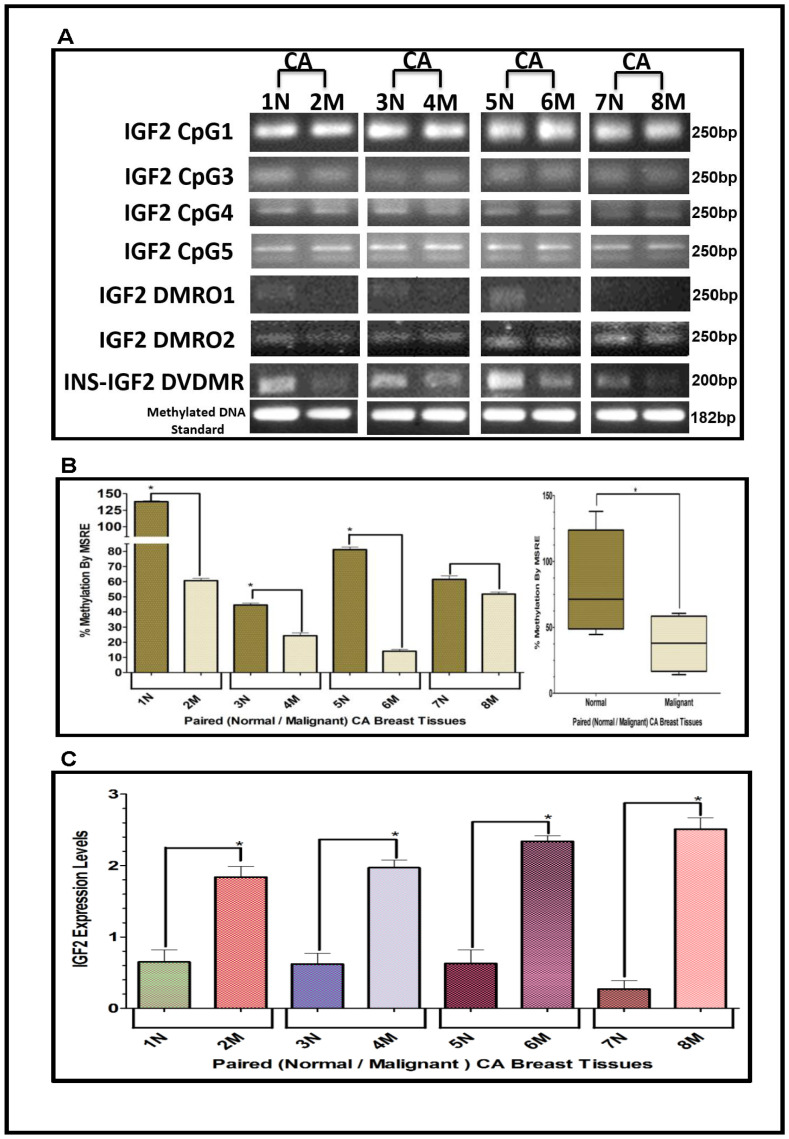
(**A**–**C**) EtBr Agarose gel of IGF2 CpG islands and DMRO’s and Bar graphs of % methylation and IGF2 protein levels in paired normal/malignant breast tissues from CA women. (A) shows one of two ethidium bromide stained agarose gels of bisulphite g-DNA fragments produced by conventional PCR amplification of the CpG and DMRO regions of the IGF2 gene in paired breast cancer tissues from Caucasian American (CA) women. (B) shows a comparative bar graph of the methylation of the INS-IGF2 DVDMR in paired tissues from CA women. Average % MSRE Methylation shown by Box and Whisker Plots. (C) shows a bar graph of the IGF2 levels in the paired breast tissues from CA women assessed by the RPPA assay. The Breast Cancer tissue samples details are shown in Table 2. ^*^symbol denotes paired samples (Normal/Malignant) from same patient.

**Figure 5:**
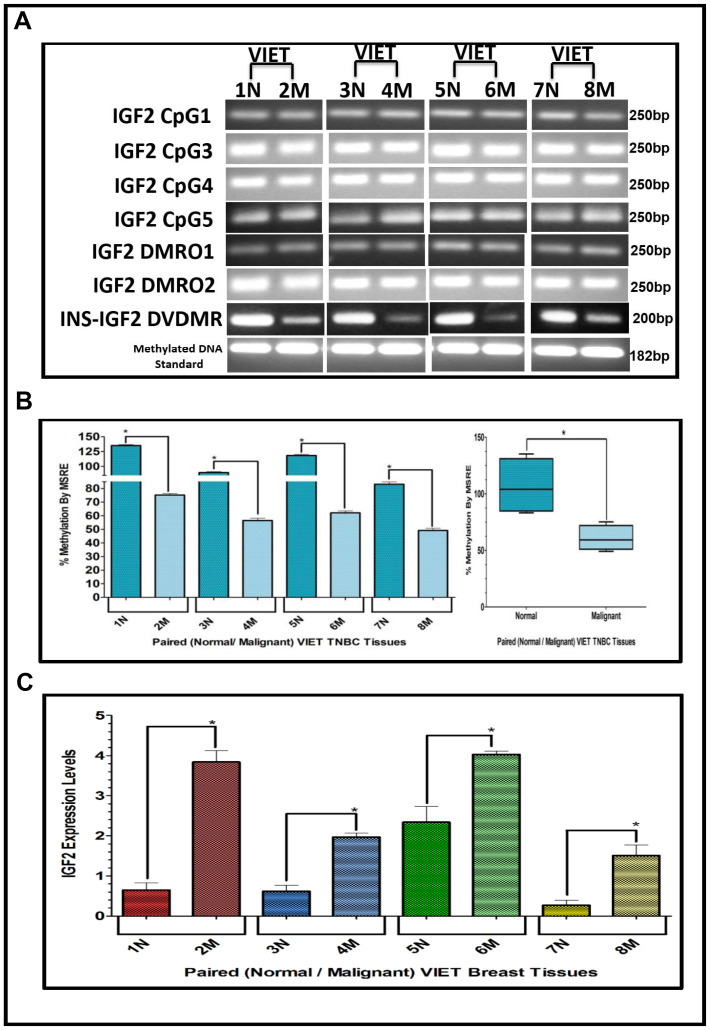
(**A**–**C**) EtBr Agarose gel of IGF2 CpG islands and DMRO’s and Bar graphs of % methylation and IGF2 protein levels in paired normal/malignant breast tissues from VIET women. (A) shows an ethidium bromide stained agarose gel of bisulphite g-DNA fragments produced by PCR amplification of the CpG and DMRO regions of the IGF2 gene in paired breast cancer tissues from Vietnamese women (VIET); (B) Shows a comparative bar graph of the methylation of the INS-IGF2 DVDMR in paired tissues VIET by Methyl Sensitive Restriction Enzyme (MSRE) qPCR; Average % MSRE Methylation shown by Box and Whisker Plots. (C) Bar graph of the IGF2 levels in paired breast tissues assessed by RPPA assay. The TNBC tissue samples details are shown in Table 2. ^*^symbol denotes paired samples (Normal/Malignant) from same patient.

The results shown above were obtained from studies performed in BC cell lines. To determine if we could corroborate these results in paired (Normal/Malignant) tissue samples obtained from BC patients, we evaluated the methylation status of IGF2 CpG islands (CpG1, CpG3, CpG4, CpG5), DMR1, DMR2, and the newly identified INS-IGF2 DVDMR from twelve paired Normal-Malignant breast tissues (4 AA, 4 CA and 4 VIET). A list of the paired breast samples used in Figures 3–5 is presented in Table 2 and includes receptor status (i. e., ER, PR, Her2) as well as age and race. Ethidium bromide-stained agarose gels showing bisulphite g-DNA fragments produced by PCR amplification of the CpG and DMR regions of the IGF2 gene and the INS-IGF2 DVDMR obtained from paired breast tissues (Normal/Malignant) from AA (Figure 3), CA (Figure 4) and VIET (Figure 5) patients are shown in panel A of each figure. No significant changes in the methylation pattern for any of the IGF2 CpG islands or DMR were observed in any of the three groups examined. In contrast, the INS-IGF2 DVDMR (257bp) is hypomethylated in malignant tissues as compared to the paired normal tissues in all three ethnic groups as shown in the agarose gels included in Figures 3A, 4A and 5A. Universal Methylated Human DNA standard (182bp) and control primers were used to check the efficiency of bisulfite-mediated converted DNA by conventional PCR methods.

We next examined the methylation patterns of the INS-IGF2 DVDMR for the same tissue samples analyzed in Figures 3A, 4A and 5A. Normal paired breast samples exhibited hypermethylation while malignant paired breast tissues displayed hypomethylation patterns for INS-IGF2 DVDMR in (Normal/Malignant) paired AA (Figure 3B), CA (Figure 4B) and VIET (Figure 5B) breast tissue samples. As observed in the BC cells analyzed, the DVDMR was also hypomethylated in the malignant breast tissues, whereas it was hypermethylated in normal breast tissues. The Box and Whisker plots shown for each figure compare the combined results of percent methylation of normal tissues to the percent methylation of malignant tissues in each group (Figures 3B, 4B and 5B). The significant difference (^*^ *p <* 0.05) in DVDMR methylation between normal vs malignant breast samples in all three groups indicates that methylation changes in the INS-IGF2 DVDMR are associated with IGF2 expression and BC progression.

### IGF2 protein in breast cancer samples using reverse phase protein array (RPPA)

Since methylation of the INS-IGF2 DVDMR in the BC cells was inversely correlated with IGF2 protein levels, we analyzed the paired breast tissues by reverse phase protein array (RPPA) to assess if IGF2 protein levels were also inversely correlated with the methylation of the DVDMR. Data shown in Figures 3C, 4C and 5C represents the levels of IGF2 protein in paired Normal/Malignant breast tissues from AA, CA and VIET, respectively. Higher IGF2 levels were detected in malignant tissues as compared to their paired normal tissue in all three groups. Thus, the IGF2 levels in the paired tissues are inversely correlated to the percent methylation levels detected in the INS-IGF2 DVDMR region. Hypomethylation of the DVDMR region leads to increased IGF2 protein levels in the malignant tissues while hypermethylation of the DVDMR lead to lower IGF2 protein levels in all normal breast tissues. These observations are significant because they suggest that similar mechanisms of IGF2 gene methylation occur *in vitro* (BC cells) and *in vivo* (paired breast tissues).

### Methylation of the INS-IGF2 DVDMR in TNBC tissues

The results presented above show that the INS-IGF2 DVDMR is significantly hypermethylated in normal breast tissues when compared to their respective paired malignant tissues. The analysis of paired breast tissue samples shown in Figures 3 and 4 was done in tissues with combined receptor status; ER+ or ER-, PR+ or PR-, and HER2+ or HER2-. Figure 5 shows the analysis of breast samples obtained from VIET breast cancer patients (only TNBC).

**Figure 6:**
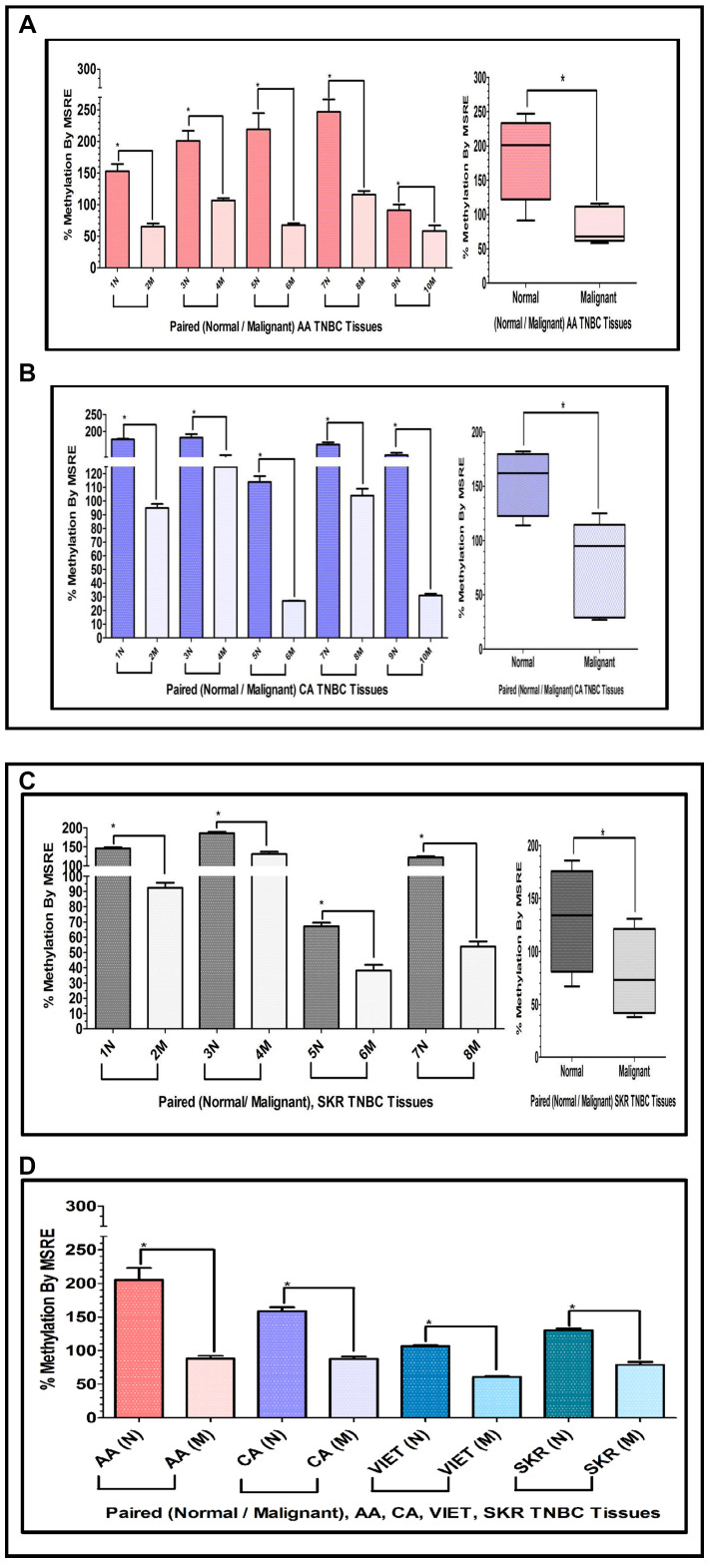
(**A**, **B**) Comparative bar graphs representing the methylation status of the INS-IGF2 DVDMR in paired breast cancer tissues exclusively obtained from TNBC tumors. (A, B) Shows comparative bar graphs representing the methylation status of the INS-IGF2 DVDMR assessed by MSRE qPCR in paired TNBC tissues from AA women (A) and CA women (B). Box and Whisker Plots next to each bar graph show the average (mean ± SEM) of the % MSRE in AA and CA. The TNBC tissue Samples details are listed in Table 3. ^*^symbol denotes paired samples (Normal/Malignant) from same patient. (**C**, **D**) Comparative bar graphs representing the methylation status of the INS-IGF2 DVDMR in paired TNBC tissues from SKR women (C), Average % MSRE Methylation shown by Box and Whisker Plots for SKR TNBC women are compared and show in (D). The TNBC tissue Samples details are shown in Table 1 for VIET and AA, CA and SKR in Table 3. ^*^symbol denotes paired samples (Normal/Malignant) from same patient.

To determine if similar results could be obtained assessing tissues that are exclusively TNBC, we extracted DNA from paired TNBC samples obtained from the Cooperative Human Tissue Network (CHTN) (AA, CA). We also analyzed the DNA obtained from TNBC tissues of South Korean (SKR) patients obtained from Dr. Dae-Kwang Kim (Department of Medical Genetics, School of Medicine, Republic of Korea). Figure 6 shows comparative bar graphs representing the methylation status of the INS-IGF2 DVDMR assessed by MSRE qPCR in paired TNBC tissues (Table 3) from AA women (Figure 6A), CA women (Figure 6B) and SKR women (Figure 6C). Next to each MSRE Methylation graph of the pair samples (Figure 6A–6C) you’ll see a Box and Whisker Plot that shows the average (mean+/-SEM) of the paired samples MSRE in each ethnic group. Figure 6D shows all the Box and Whisker Plots comparing the methylation status of the INS-IGF2 DVDMR from paired TNBC samples of all four ethnic groups (AA, CA, VIET and SKR).

**Table 3:** Paired tissues from AA, CA and SKR TNBC patients samples

Sample	ID	Receptor status	Age	Ethnic group
Normal	1M	ER-/PR-/Her2-	59	AA
Tumor	2N			
Normal	3M	ER-/PR-/Her2-	46	AA
Tumor	4N			
Normal	5M	ER-/PR-/Her2-	36	AA
Tumor	6N			
Normal	7M	ER-/PR-/Her2-	42	AA
Tumor	8N			
Normal	9M	ER-/PR-/Her2-	60	AA
Tumor	10N			
Normal	1M	ER-/PR-/Her2-	43	CA
Tumor	2N			
Normal	3M	ER-/PR-/Her2-	91	CA
Tumor	4N			
Normal	5M	ER-/PR-/Her2-	63	CA
Tumor	6N			
Normal	7M	ER-/PR-/Her2-	72	CA
Tumor	8N			
Normal	9M	ER-/PR-/Her2-	63	CA
Tumor	10N			
Normal	1M	ER-/PR-/Her2-	44	SKR
Tumor	2N			
Normal	3M	ER-/PR-/Her2-	44	SKR
Tumor	4N			
Normal	5M	ER-/PR-/Her2-	63	SKR
Tumor	6N			
Normal	7M	ER-/PR-/Her2-	41	SKR
Tumor	8N			

These data demonstrates that the methylation of the INS-IGF2 DVDMR results obtained from TNBC samples are similar to those obtained from paired samples with combined different ER, PR and HER2 status. Thus, the methylation pattern of the DVDMR is not dependent of the tissue receptor status. Regardless of which receptors are expressed, breast tumor samples are significantly hypomethylated when compared to paired normal breast tissues.

### DISCUSSION

The gene encoding IGF2 is extremely complex, which makes very challenging understanding its regulation. This growth factor is transcriptionally controlled by methylation of 5 different promoters and differentially methylated DNA regions (DMRs). In addition, IGF2 is inhibited and or stimulated by several hormones, stress, nutrition, tumor suppressors and oncogenes [23, 29, 30]. The chromatin structure of the IGF2 gene and its regulatory elements involves various long linear non-coding RNA and microRNAs [18–22]. Rotwein’s recent review on the complexity of the IGF2 gene further expands on the subject of how the intricacies of this gene are not even displayed in current public databases [29]. As we learn more about how IGF2 functions in health and disease, it becomes clear that a thorough understanding of this gene’s actions is needed to address its potential clinical use.

Methylation is an important cellular process in the regulation of IGF2. Each one of the IGF2 gene five promoters generate distinct transcripts which vary by tissue type and cellular developmental stages [11, 12, 29]. In human tissues, the IGF2 gene is controlled by at least two differentially methylated regions (DMRs). One DMR is located upstream of the IGF2 promoters (IGF2 DMR) and the second DMR is located upstream of the neighboring non-coding H19 gene (H19 DMR) [13]. Similarly, there are multiple repeats of CpG islands that also control the transcription of the different IGF2 variants. These CpG repeats are also modified by methylation and this modification determines which IGF2 promoter gets activated. Thus, methylation of the IGF2 DMRs controls the transcription of the IGF2 gene.

Abnormal methylation of IGF2 leads to various metabolic disorders like BC, pancreatic cancer, diabetes and endocrine related disorders [24–26]. Changes in the IGF2 gene methylation varies among the promoters, and differential IGF2 gene methylation patterns lead to distinct clinical features in BC and other diseases [31–33]. For example, there is a differential methylation of the IGF2 exon 9 CpG cluster, which is hypomethylated in tumor tissues but hypermethylated in normal paired tissues [28].

Due to the complexity of the regulation of IGF2 expression, understanding the changes in IGF2 gene methylation will provide valuable insights into how IGF2 promotes BC progression. To address this need, we focused the present study on analyzing the DNA methylation patterns of the IGF2 gene in paired (Normal/Tumor) tissues obtained from BC patients from different ethnic groups. DNA methylation pattern of the IGF2 gene was also analyzed in several BC cell lines to determine if there was a correlation between methylation in the IGF2 gene and nearby regions with the levels of IGF2 mRNA and protein. Initially, we characterized the IGF2 P1-P4 promoter transcript variance in four TNBC cell lines. Since IGF2 transcripts are differentially expressed, we aimed to determine if there was a preference for the usage of a particular promoter in BC and how it may correlate with total IGF2 mRNA and protein.

The IGF2 transcripts detected in the BC cell lines originated from promoters P1, P3 and P4. ln a recently published analysis of the human IGF2 gene, data from the Exome Aggregation Consortium showed that the IGF2 P1 promoter was only expressed in normal liver out of 38 normal tissues examined [29]. In contrast, our data identified transcripts derived from P1 in all four BC cell lines analyzed. It is interesting that the IGF2 transcripts generated from P1 were only expressed in normal liver out of 38 normal tissues examined. IGF2 is a potent mitogen that is required for normal fetal development and cell differentiation [2]. Nevertheless, at birth, the expression of IGF2 in circulation is significantly reduced in most species, but not in humans, were detectable IGF2 produced by the liver remains in circulation [34]. Recent studies have shown that IGF2 is a key player in driving regeneration and hepatocyte repopulation in the liver [35]. IGF2 is also essential in regulating stem cell activity in adult tissues [36]. Thus, IGF2 transcripts generated from P1 may preferentially respond to factors activated by rapidly growing cells such as hepatocytes and cancer cells.

No IGF2 mRNAs were identified from the P2 promoter in any of the cells analyzed in our study. Similarly, no IGF2 transcripts generated from P2 were identified in any of the 38 human tissues, including normal mammary tissue [29]. While discussing the Consortium data, the author proposes the possibility that IGF2 transcripts generated from P2 may determine the activation of the INS-IGF2 transcripts. The INS-IGF2 locus is within the larger region that encompasses the IGF2 gene and represents part of the DNA methylation analysis characterized in the present study. This region generates two alternatively spliced read-through transcript variants which align to the INS gene in the 5′ region and to the IGF2 gene in the 3′ region. One transcript is predicted to encode a protein which shares the N-terminus with the INS protein but has a distinct and longer C-terminus, whereas the other transcript is a candidate for nonsense-mediated decay. Similar to IGF2, the INS-IGF2 gene is imprinted and is paternally expressed in the limb and the eye [27].

We identified a new region with variable methylation within the INS-IGF2 locus, between exons 3 and 4, which we named INS-IGF2 DVDMR. This DMR consists of 257bp located in chromosome 11 and spans between bps 2148098 – 2148354 (GRCh38/hg38). Hypomethylation of the INS-IGF2 DVDMR correlated with the increase of both INS-IGF2 transcripts in all four cell lines. Unexpectedly, hypomethylation in this region also correlated closely with increased IGF2 mRNA and protein levels in all four cell lines. Interestingly, this novel observation regarding the correlation of hyper or hypo methylation of the INS-IGF2 DVDMR to decreased or increased IGF2 levels, respectively, was also observed in the paired breast tissue samples analyzed in our study.

Our data also showed that the methylation profile of the INS-IGF2 DVDMR in paired breast tissues was distinct and could differentiate malignant breast tissue (hypomethylated) from normal adjacent breast tissue which was hypermethylated. This methylation pattern was observed in TNBC tissues and in all paired breast tissues examined regardless of ER, PR and HER2 receptor status. Similarly, the same methylation pattern in normal vs malignant breast tissue was observed in paired tissues from four different ethnic groups examined. These results suggest that methylation of the INS-IGF2 DVDMR is a key regulator of IGF2 expression in BC.

IGF2 promotes cell proliferation, inhibit apoptosis and stimulate transformation of BC cells [37, 38]. IGF2 is also highly expressed in BC patients and “free” circulating IGF2 levels in humans are significantly correlated with breast tumor size [8]. Transgenic animal models with increased IGF2 expression show a significant increase in BC that develops at an early age and it is more aggressive [39–43]. Our team has previously identified IGF2 as an important biological factor contributing to higher BC mortality among AA women [44, 45]. Of significance, we demonstrated that IGF2 levels inhibited or stimulated mitochondrial proteins in BC cell lines, preventing cell death and inducing chemoresistance [46, 47]. These studies also showed that mitochondrial proteins were significantly correlated with IGF2 levels in tissues from BC patients [44]. Since mitochondria are important targets of chemotherapy, IGF2 expression by BC tumors may confer mitochondrial protection, thereby, inducing chemoresistance and effectively reducing clinical treatment efficacy. We propose that IGF2 is a critical biological factor in BC that contributes to the development of chemoresistance and may increase BC mortality. Thus, IGF2 represents a potential therapeutic target to decrease chemoresistance and improve survival among BC patients.

In summary, the present study shows that IGF2 expression in BC cells and in paired Normal- Malignant breast tissues are determined by the methylation of a novel region in the INS-IGF2 locus. We are currently studying the mechanisms underlying the methylation of the INS-IGF2 and how they control IGF2 expression. Upregulation of IGF2 in terms of the methylation patterns of the DVDMR may have an important function in the tumorigenesis of the breast. In conclusion, we propose that the INS-IGF2 DVDMR may be a useful tool to identify women at risk of developing a more aggressive BC disease.

## MATERIALS AND METHODS

### Tissues and cell lines

The cell lines used in this study were obtained from the American Type Culture Collection (ATCC). The tissue samples for AA and Caucasian CA) patients were obtained from the Cooperative Human Tissue Network (CHTN) and the South Korean (SKR) TNBC gDNA samples were obtained as gift samples from Dr. Dae-Kwang Kim, Department of Medical Genetics, School of Medicine, Institute for Medical Genetics, Keimyung University, Hanvit Institute for Medical Genetics, 2800 Dalgubeoldaero, Dalseo-Gu, Daegu 704-701, South Korea. The tissue samples from the Vietnamese (VIET) TNBC patients were obtained from ILSbio (Integrated Laboratory Services-Biotech, 100 Radcliffe Drive, Chestertown, MD 21620 USA).

### Sodium bisulphate and methyl PCR array

One μg of gDNA was extracted using PureLinkTM Genomic DNA Mini kit from Invitrogen (Camarillo, CA, USA) from breast tissue samples of AA, CA, VIET, and SKR BC patients, and from BC cell lines. The sodium bisulphate gDNA conversion was performed using EZ DNA Methylation-Lightning™ Kit from Zymo Research (Irvine, CA) which converts unmethylated cytosine nucleotides into uracil nucleotides, while methylated cytosine remains unaltered. PCR further converted uracils into thymines and methylated cytosines into cytosines using a probe specific methylation primer and HotStar DNA polymerase from (Qiagen) to amplify CpG, IGF-2 DMR and INS-IGF2 DVDMR PCR products using 58° C as the annealing temperature. The methyl specific primers for CpG and DMR, listed in Table 1, were used for amplifying the methyl specific PCR products, which were electrophoresed into a 2% agarose gel and stained with ethidium bromide to visualize the difference between the hypo- and hyper-methylation patterns in normal and tumor samples as previously described [29, 30].

### RNA, cDNA, qPCR

RNA was isolated using TRI REAGENT (Zymo Research, Inc.) and treated with DNase. Bio Rad’s iScript Synthesis Kit was used for cDNA synthesis. Specific primers for the IGF2 and INS-IGF2 short and long transcripts were designed with intron spanning assay primer designing tools. Methylation primers were designed using the MethPrimer |Tools and Databases from the Li Lab (https://www.urogene.org/methprimer2/) as shown in Table 1. QPCR was performed using the CFX96 and CFX1000 touch thermal cyclers. The IGF2 and INS-IGF2 mRNA transcript fold expression results were analyzed using the CFX Manager software Version 1.0.

### Restriction digestion and methylation real-time PCR assay

To isolate DNA from BC tissues and cell lines, we used the PureLinkTM Genomic DNA Mini kit from Invitrogen (Camarillo, CA, USA). 1mL syringe with 27 1/2-gauge needle was used to lyse the homogenized cell and tissue samples. The methylation PCR assay was performed utilizing the HhaI and McrBC restriction enzymes from the New England Biolabs, (MA, USA). Primers were chosen to flank both, the MSRE and MDRE restriction sites within the region of interest of the INS-IGF2 gene. To design the primers, we used the Primer3 software (http://www.bioinfo.ut.ee/primer3-0.4.0/) according to standard principles for qPCR outlined in the QuantiTectTM SYBR Green PCR Handbook from Qiagen. Primers can also be used in parallel to Sham and Methylated fractions collected. Each of these factors contributes to the successful generation and interpretation of methylation results. MSRE analysis facilitates the quantitation of DNA methylation in one combined step with qPCR assay. These restriction enzymes are unable to cleave methylated-cytosine residues, thus, only methylated DNA remains intact and can be quantified. The procedure used a triplicate set for each sample MSREs (HpaII) and sham templates. The XXXCt versus percent methylation relationship is derived from the basic principle that each successive round of PCR amplification results in approximately a two-fold increase in the amount of product. Thus, XXXCt of 1.0 indicates that 50% of the template has been cleaved, 2.0 equals 75% cleavage.

### Reverse phase protein array for quantifying IGF II levels (RPPA)

RIPA buffer was used to homogenize the samples, and protease inhibitors were added to the buffer to prevent protein degradation. For each sample, whole lysates, neat plus four serial dilutions (dilution factor 0.8) and one buffer control, were pipetted into V-shaped ABgene 384-well plates (Thermo Fisher Scientific, Rockford, IL, USA). Samples were printed in triplicate onto nitrocellulose-coated glass slides (Supernova slides, Grace Bio-Labs, Inc., Bend, OR, USA), using an Aushon BioSystems 2470 Arrayer (Aushon BioSystems, Billerica, MA, USA) with 185 mm pins and a single touch. Slides were stored at 4° C until use. Briefly, slides were incubated in blocking solution (3% BSA (Sigma-Aldrich Co, Saint Louis, MO, USA) in TBST (Tris-buffered saline, 0.1% Tween 20) for 4 h, followed by overnight incubation at 4° C with the primary antibody, IGF2 monoclonal antibody from Amano. Slides were washed three times for 5 min in TBST, followed by the incubation with Fluorescence Alexa Fluor 700 anti mouse (Invitrogen) secondary antibody (1:1000) for 90 min at room temperature. Slides were washed three times for 5 min with TBST and dried for 20 min at 30° C. All the steps during and after incubation with secondary antibody were performed in the dark. Antibody-stained slides were scanned using a Scan Array Express HT Microarray Scanner (PerkinElmer, Inc. Boston, MA, USA). The intensity of each spot was quantified using the ScanArray Express software (PerkinElmer, Inc., Boston, MA, USA). Antibody signal for each spot was normalized to the corresponding signals from staining a different slide with the general protein stain SyproRuby, following the manufacturer’s instructions (Invitrogen, Camarillo, CA, USA).

### Statistical analysis

Statistical analysis was determined by using one-way ANOVA and the Wilcoxon Signed Rank Test and paired *T*-Test, which were used for comparing the paired Normal/Malignant samples in GraphPad Prism 5. Values are expressed as the mean ± SEM of at least 3 separate experiments done in triplicate. A *P* value < 0.05 was considered significant. Experiments depicted in Figures 3A, 4A and 5A (agarose gels) were not subjected to statistical analysis.

## Abbreviations

AA: African American
CA: Caucasian American
DMR: Differentially Methylated Region
VIET: Vietnamese
SKR: South Korean
TNBC: Triple Negative Breast Cancer
IGF2: Insulin-Like Growth Factor2
INS-IGF2: Insulin-Insulin-Like Growth Factor2
MSRE: Methyl Sensitive Restriction Digestion
